# 

**Published:** 1882-10

**Authors:** 


					﻿ATTENTION!!!
The Central Ohio Homoeopathic Medical Society has decided to offer a prize
for provings. The design is to secure accurate re-provings of some partially
tested remedies. The prize will be given to the physician who may present the
most valuable proving. All homoeopathic physicians and medical societies
are invited to enter the contest. The prize to be given will be Allen’s Ency-
clopaedia of Pure Materia Medica, or its equivalent in homoeopathic publica-
tions, to be selected by the winner. The award will be made by three experts
in materia medica not connected with the Society. Any who desire to con-
duct such work upon themselves, their patients or friends, are requested to send
to Dr. John C. King, Circleville, Ohio, for circulars containing further informa-
tion. It is hoped that members of our school who desire a more accurate materia
medica and who are anxious for re-provings, conducted upon scientific princi-
ples (see circular), will respond to this call. All work presented will be freely
made the property of the profession or promptly returned to the author. Any
one of the three specified drugs may be selected. To obtain full particulars
send for circular. Respectfully submitted.
Committee on Provings—John 0. King, M. D., Sec’y, Circleville, O.; E. R.
Eggleston, M. D., Mt. Vernon, O.; J. W. Clemmer, M. D., Columbus, O.
NOTICE. '
All articles for publication, books for review, business communications, etc.,
must be sent to Editor, 2109 Chestnut Street, Philadelphia.
CONTRIBUTORS:
Drs. H. C. Allen, T. F. Allen, Edward Bayard, J. B. Bell, E. W. Berridge,
Geo. H. Clark, As C. Cowperthwaite, Wm. J. Guernsey, W. A. Hawley, j
John Hall, W. M. James, W. H. Jenny, Ad. Lippe, C. Lippe,
Malcolm MacFarlan, C.- F. Nichols, C. Pearson, T. F. Pom-
eroy, Leila A. RenDell, C., Carleton Smith, P. P. Wells, .
Wm. P. Wesselhoeft, David Wilson .(London),
T. P. Wilson, and many others.
Authors' of accepted papers are furbished with copies of the
number in which their articles appear; application for such copies
to be made when sending papers. Any irregularity in the receipt
of this Journal should be promptly reported to Editor.
				

